# Association of polymorphisms in ADAMTS-5 gene with the susceptibility to knee osteoarthritis: A protocol for systematic review and meta-analysis

**DOI:** 10.1097/MD.0000000000028188

**Published:** 2021-12-10

**Authors:** Tingting Deng, Yue Zhou, Kaixin Zhang, Zhibin Dong, Jingwen Zhang, Xinwei Lv, Shuai Song, Yuxia Ma

**Affiliations:** a Shandong University of Traditional Chinese Medicine, Jinan, Shandong, China; b Affiliated Hospital of Shandong University of Traditional Chinese Medicine, Jinan, Shandong, China.

**Keywords:** ADAMTS5, KOA, meta-analysis, protocol, systematic review

## Abstract

**Background:**

To systematically review literature evidence to discover the association of ADAMTS5 (A Disintegrin And Metalloproteinase with Thrombospondin-like motifs 5) gene polymorphisms and the risk of developing KOA (knee osteoarthritis).

**Methods:**

We systematically searched the related randomized controlled trials in 4 databases from inception to August 2021, including the Embase, Web of Science, PubMed, and CNKI (Chinese National Knowledge Infrastructure) databases. No language and publication status restrictions. Two reviewers will independently screen all included studies, and the meta-analysis will be conducted using the Review Manager (RevMan 5.3, Cochrane Collaboration, Nordic Cochrane Center, Copenhagen, Denmark).

**Results:**

The gathered evidence suggests that there may be a close relationship between the SNP in the ADAMTS5 gene and KOA development. This study will provide a high-quality and convincing evaluation of the treatment of KOA from the consideration of ADAMTS5 gene and will be published in a peer-reviewed journal.

**Conclusion:**

ADAMTS5 polymorphism is likely an important risk factor for the development of KOA. Our study will provide a more accurate treatment method for the treatment of KOA.

**Trial registration number:**

CRD42021276317

## 1 Introduction

KOA is a clinically common degenerative osteoarthritis, with a worldwide incidence of 44% to 70% in middle-aged and elderly people over 55 years old.^[1]^ Current confirmed risk factors of KOA include oxidative stress, inflammatory factors, vitamin D, obesity, etc,^[2]^ among which the excessive degradation of chondrocyte extracellular matrix and cartilage degeneration induced by inflammatory factors are considered to be the main factors causing KOA.^[3]^ The detection of biomarkers of KOA is a hot spot and frontier of current research. The detection of various biomarkers in the occurrence and development of KOA can be used for early diagnosis before structural abnormalities of cartilage tissue appear, so it has an extremely important clinical application value. The pathological changes of KOA are mainly chondrocyte hypertrophy, apoptosis, and changes of chondrocyte extracellular matrix. In this complex process, dozens of biomarkers are related to it, but only a few biomarkers have been revealed to have important diagnostic value of KOA.

In the process of the occurrence and development of knee osteoarthritis, the severity of KOA mainly depends on the integrity of articular cartilage, and whether the integrity can be maintained is mainly determined by the proteoglycan and collagen fibers in articular cartilage. Polysaccharides are the weight of the matrix of articular cartilage. It, together with collagen lattice and chondrocytes, constitutes articular cartilage. In the early stage of arthritis, due to imbalance of synthesis and decomposition of cartilage matrix, often accompanied by the reduction of proteoglycan. There are 19 types of chondroproteoglycan known to date, among which ADAMTS-5 have significantly higher degradation activity than other types.^[4]^ Both in explants of human OA cartilage and molecular biology experiments have shown that decreased expression of ADAMTS-5 reduced the aggrecan loss stimulated from cartilage explants with inflammatory cytokines (tumor necrosis factor-a and oncostatin M).^[5,6]^ ADAMTS-5 plays a major role in the degradation of chondroproteoglycan, which is an important cause of degeneration and injury of articular cartilage. ADAMTS-5 is a promising target for the identification of knee osteoarthritis (KOA). To define the role of ADAMTS-5 in the progression of KOA, we performed the current meta-analysis.

## 2 Materials and methods

### 2.1 Study registration

This systematic review has been registered in PROSPERO (CRD42021276317) and will be carried out in accordance with the preferred reporting project declared by the systematic review and meta-analysis protocols 2015.^[7]^

### 2.2 Inclusion criteria for study selection

#### 2.2.1 Types of studies

This study will provide a high-quality and convincing evaluation of the treatment of knee osteoarthritis from the consideration of ADAMTS gene and will be published in a peer-reviewed journal. There are no language or publication restrictions. Excluding non-RCTs, reviews, experimental studies, clinical case reports, and animal research literature

#### 2.2.2 Participants

Patients diagnosed with KOA will be included, regardless of age, sex, education or race. The diagnosis of KOA includes Chinese or international diagnostic criteria.^[8,9]^

#### 2.2.3 Types of interventions

The level of ADAMTS5 mRNA in articular cartilage of KOA patients was detected by real-time quantitative PCR in the treatment group. The control group had no signs or symptoms of osteoarthritis or knee joint diseases (pain, swelling, tenderness, or restriction of movement).

#### 2.2.4 Outcomes

The following information was collected from each study: publication year, country, ethnicity, specimen source, gender and age, case and control numbers, period of KOA, and the expression of ADAMTS-5 in synovial fluid, including genotyping, genotype distributions, and relevant SNP polymorphism.

### 2.3 Search strategy

This article follows the criteria of the Preferred Reporting Items for Systematic Reviews and Meta-Analyses, with relevant research searched up to August 2021, including the Embase, Web of Science, PubMed, and Chinese National Knowledge Infrastructure (CNKI) databases. The keywords used for search were as follows: “A disintegrin and metalloproteinase with thrombospondin motifs 5” or “ADAMTS-5” or “ADAMTS5” or “ aggrecanase-2” or “ADAMTS 5 gene,” “Knee osteoarthritis” or “osteoarthritis of knee” or “KOA” or “knee OA” or “knee joint OA.” The search strategy in PubMed is as follows:

1. #1 Search: ((((A disintegrin and metalloproteinase with thrombospondin motifs 5[MeSH Terms])) OR (ADAMTS-5[Title/Abstract])) OR (aggrecanase-2; [Title/Abstract])) OR (ADAMTS 5 gene[Title/Abstract])
2. #2 Search: (((((Knee osteoarthritis[MeSH Terms])) OR (osteoarthritis of knee[Title/Abstract])) OR (KOA[Title/Abstract])) OR (knee OA[Title/Abstract])) OR (knee joint OA[Title/Abstract]);
3. #3 Search: (((((((((clinical trials, randomized[MeSH Terms]) OR (random allocation
4. [MeSH Terms])) OR (RCT[Title/Abstract])).
5. #4 #1 and #2 and #3

### 2.4 Data collection and analysis

#### 2.4.1 Selection of studies

**Figure 1:**
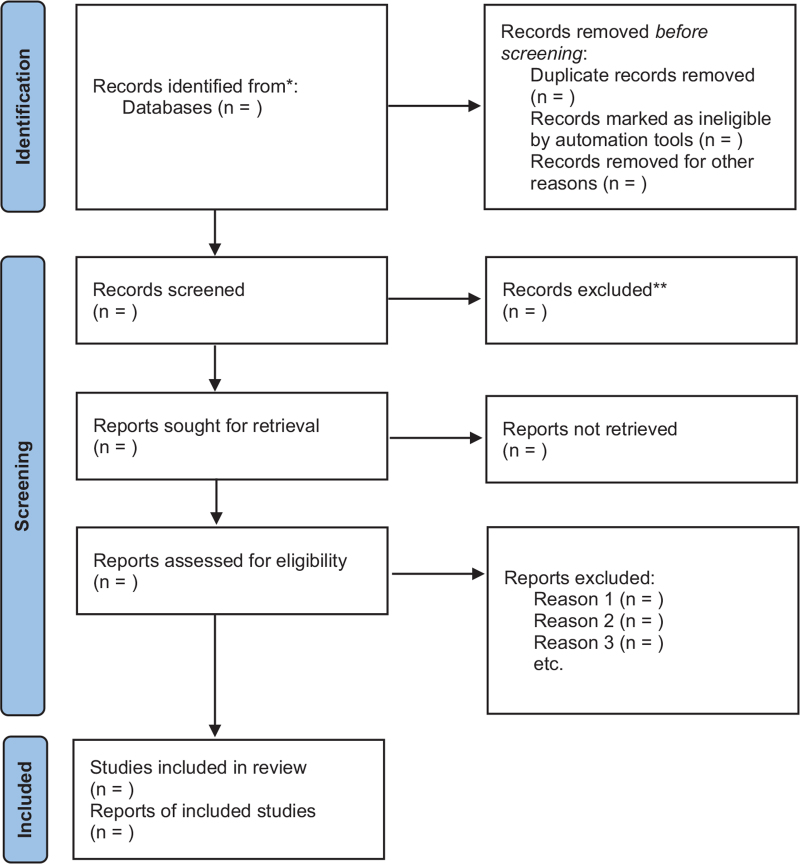
The PRISMA flow diagram.

The search strategy and study selection were independently performed by 2 researchers on a database search, and the final research choices were agreed upon. Two researchers independently evaluated the same article to determine their eligibility for inclusion and resolved differences through consensus. The filtering process is shown in Figure 1.

#### 2.4.2 Data extraction and management

Two reviewers will be responsible for the extraction and management of data according to the retrieval strategy, including study title, journal, year of publication, name of first author, general information, study design, period of KOA, and the expression of ADAMTS-5 in synovial fluid, including genotyping, genotype distributions, and relevant SNP polymorphism, adverse events, etc. If there is any disagreement between the 2 reviewers during the data extraction process, the panel will jointly arbitrate and make a decision.

#### 2.4.3 Dealing with missing data

If information is missing or unclear, we will try to contact the corresponding author for more detailed information. If it fails, we will analyze it based on the available data.

#### 2.4.4 Assessment of risk of bias

The sixth edition of the Cochrane Handbook for systematic reviews of interventions will be used to assess the broad categories of biases in the included study. Bias will be evaluated from the following 7 aspects: random sequence generation, allocation hiding, blindness of participants and personnel, blindness of result evaluation, incomplete outcome data, selective reports and deviations from other sources. These studies will be divided into low-risk, high-risk, and ambiguous risks. Inconsistencies will be resolved through discussions with other reviewers.

#### 2.4.5 Assessment of quality of evidence

All studies used the Newcastle-Ottawa Scale (NOS) assessment scale^[10]^ for evaluation. Relevant data was extracted independently by 2 reviewers (Tingting Deng and Yue Zhou), disagreement was resolved through discussion, and the third reviewer (Yuxia Ma) was dealt with if necessary. The scale includes 9 items, covering 3 dimensions. The study is awarded 1 point for each item satisfied. The NOS score ranges from 0 to 9, with higher scores indicating better quality. In this study, a score ≥ 6 was considered better quality.

#### 2.4.6 Measures of treatment effect

Standardized mean differences (SMDs) and 95% confidence intervals (CIs) were used to calculate the difference of ADAMTS-5 gene between KOA and control group.

#### 2.4.7 Assessment of heterogeneity

Q-statistic (*P* < .05) and *I*^*2*^ statistics (*I*^*2*^>5 0%) were used to evaluate the heterogeneity among studies.

#### 2.4.8 Assessment of reporting bias

If more than 10 studies are included in the meta-analysis, a funnel chart will be used to detect reporting deviations.^[11]^

#### 2.4.9 Data synthesis

We used the Review Manager (RevMan 5.3, Cochrane Collaboration, Nordic Cochrane Center, Copenhagen, Denmark) for statistical analyses. Standardized mean differences (SMDs) and 95% confidence intervals (CIs) were used to calculate the difference of ADAMTS-5 gene between KOA and control group. When some studies reported using the of results of the median of the first and third quartiles (M (P_25_, P_75_)), approximation methods were used to calculate the mean and standard deviation (*X* ±*S*)^[12]^;X=(P_25_+M+P_75_)/3 and S = (P_75_–P_25_)/1.35. Besides, all standard errors of the correlation coefficient (SE) were calculated as follows;^[13]^ SE =√((1−r)/(n−1))^2^ to combine the *r* of the random-effect model. Q-statistic (*P* < .05) and *I*^*2*^ statistics (*I*^*2*^>5 0%) were used to evaluate the heterogeneity among studies. In individual studies, we used the Cochrane Collaboration's tool to assess the risk of bias. *P* < .05 indicates statistical significant.

#### 2.4.10 Subgroup analysis

If feasible, we will conduct subgroup analysis based on different interventions, controls, treatment duration and outcome indicators.

#### 2.4.11 Sensitivity analysis

A sensitivity analysis will be conducted to investigate the robustness of the research conclusions. Methodological quality, sample size, and the impact of missing data will be included. Therefore, the impact of low-quality research on the overall results will be assessed.

#### 2.4.12 Ethics and dissemination

This work does not require related ethical review, because there is no data related to individual patient or animal information. Our research results will be shared and displayed through peer-reviewed journals.

## 3 Discussion

KOA is the most common degenerative joint disease, and its pathological changes involve cartilage, subchondral bone, meniscus, slide membrane, ligament and muscle, etc., is an important cause of knee joint swelling, pain, stiffness, limited activity, seriously affect patients’ healthy body and daily life, the incidence of this disease is much higher than other parts of OA.^[14]^ The imbalance between ECM (the extracellular matrix) synthesis and degradation is an important feature of cartilage degradation and degeneration.^[12]^ ECM of cartilage is mainly composed of Col2 and PG,^[13]^ and 90% of PG is dominated by aggrecan. Aggrecan complex distributes in collagen network and is mainly responsible for maintaining normal physiological functions of cartilage. When aggrecan is degraded by aggrecanase, the fibrillar collagen network is damaged and exposed, and ECM is denatured to replace the once healthy functional matrix, resulting the tissue to become thin and mechanically weak.^[15,16]^ In KOA, the enzymes that play a major role in aggrecan degradation of articular cartilage are aggrecanases -- ADAMTS-4 and ADAMTS-5. In the cartilage of patients with KOA, ADAMTS-5 locates around the cluster of cloned cells. ADAMTS-5 cleaves aggrecan core protein at the aggrecanase-specific Glu373- Ala374 bond in the IGD region.^[16,17]^ The expression of ADAMTS-5 gene can be regulated by epigenetic modifications such as histone acetylation and deacetylation, and changes in methylation status of CpG sites in gene promoters. Therapies that halt or slow the deterioration of joint structure and function will address the current major need for treatment of KOA. By strictly regulating the activity of ADAMTS-5 to maintain a good balance between aggregative polysaccharides anabolism and catabolism, it is possible to slow down progressive cartilage erosion and even prevent the development of end-stage disease. To verify that ADAMTS5 single nucleotide polymorphism is the potential cause of KOA development trend, we evaluated published evidence from randomized controlled trials on the relationship between ADAMTS5 gene polymorphism and KOA susceptibility. This will help establish a personalized predictive model to predict the phenotype of knee osteoarthritis in the future, and provide a more accurate treatment method for the treatment of KOA.

## Acknowledgments

This study was supported in part by the National Natural Science Foundation of China (No. 81774402).

## Author contributions

All authors had access to the data and a role in writing the manuscript. All authors read and approved the final manuscript.

**Data curation:** Tingting Deng.

**Formal analysis:** Yue Zhou.

**Methodology:** Tingting Deng, Kaixin Zhang, Zhibin Dong and Shuai Song.

**Project administration:** Yuxia Ma.

**Resources:** Tingting Deng and Jingwen Zhang.

**Software:** Tingting Deng, Xinwei Lv.

**Visualization:** Yue Zhou.

**Writing – original draft:** Tingting Deng and Yuxia Ma.

**Writing – review & editing:** Yuxia Ma.
